# Opsoclonus-myoclonus syndrome after adenovirus infection

**DOI:** 10.1186/s40064-015-1429-1

**Published:** 2015-10-23

**Authors:** Steffen Syrbe, Andreas Merkenschlager, Matthias K. Bernhard, Jens Grosche, Uwe Gerd Liebert, Wolfgang Hirsch, Wolfgang Härtig

**Affiliations:** Department of Women and Child Health, Hospital for Children and Adolescents, University Hospitals, University of Leipzig, Liebigstr 20a, 04103 Leipzig, Germany; Paul Flechsig Institute for Brain Research, University of Leipzig, Liebigstr. 19, 04103 Leipzig, Germany; Institute of Virology, University of Leipzig, Johannisallee 30, 04103 Leipzig, Germany; Section Paediatric Radiology, Department of Imaging and Radiotherapy, University of Leipzig, 04103 Leipzig, Germany

**Keywords:** Children, Autoimmune, Ataxia, Encephalopathy, Movement disorder

## Abstract

Autoimmune and paraneoplastic movement disorders are rare in childhood. Diagnosis often relies on clinical manifestations and clinicians’ recognition. A 22-month-old girl at onset of opsoclonus-myoclonus syndrome (OMS) was followed for 8 years. Adenovirus (type C subtype 3) infection coincided with manifestation. Data on treatment, imaging and follow-up are provided. In the spinal fluid, elevated anti-rubella antibodies and oligoclonal bands were detected. An autoimmune process affecting mainly cerebellar neurons was revealed immunohistochemically. Moderately intense long-term immunosuppressive therapy resulted in a favorable clinical outcome. A video demonstrated severe OMS manifestations at onset, followed by nearly complete recovery after treatment. We describe the association of a parainfectious OMS and adenovirus infection; laboratory results indicate a non-specific humoral process affecting mainly cerebellar neurons. Our video documentation will aid to recognize this rare movement disorder and to initiate early treatment.

**Electronic supplementary material:**

The online version of this article (doi:10.1186/s40064-015-1429-1) contains supplementary material, which is available to authorized users.

## Background

The spectrum of movement disorders in childhood is diverse. Diagnosis remains often descriptive. Opsoclonus-myoclonus-(ataxia) syndrome (OMS), or dancing-eyes syndrome, is a rare encephalopathy, seen mainly in infancy with an estimated incidence per year of 0.18 per million total population (Pang et al. 2010). OMS is characterized by jerking conjugated bulbar movements, ataxia with myoclonus and psychiatric symptoms such as behavioral change. Most adult cases are of paraneoplastic origin. In about 50 % of children, OMS is associated with neuroblastoma, whereas the other cases are triggered by infections. OMS is characterized as an autoimmune disease with B- and T-cell activation. Autoantibodies binding to neuronal proteins have been described (Blaes et al. 2005; Pranzatelli et al. 2004). Empirical therapy consists of immunosuppressive agents with differing treatment strategies (Denne et al. 2006; Rostasy et al. 2006). Initiation of treatment relies on the recognition of the clinical picture. Misdiagnoses and a mean delay in correct diagnosis of 11 weeks indicate insufficient knowledge of this recognizable movement disorder among pediatricians (Tate et al. 2005).

## Case description

A formerly healthy 22-month-old girl presented for progressive gait disturbance and jitteriness since the day before. During the preceding week she was seen by her pediatrician for a febrile upper respiratory tract infection. Clinical investigation revealed mild signs of upper respiratory tract infection. Neurological examination showed a constant tremor and mild gait ataxia. Force, sensibility and deep tendon reflexes were normal, whereas pyramidal signs were absent. Magnetic resonance imaging (MRI) of the brain and electroencephalography (EEG) were performed on the first day and repeated after 2 weeks showing no pathological findings. Cerebrospinal fluid (CSF) analysis performed on day 1 revealed 12 leucocytes/µl with 83 % lymphocytes and intrathecal production of IgM and IgA antibodies. Normal values were obtained for glucose, lactate and protein. Due to contact with chickenpox 10 days prior to admittance and assumed cerebellitis, she was treated with acyclovir over 10 days. During the following 2 weeks the clinical picture deteriorated. She developed constant myoclonic jerks, severe ataxia, bobbing eye movements and extreme irritability with aggressive behavior. She was able neither to sit nor to stand, and hyperkinetic movements persisted during sleep (see Additional file 1: Video).

The clinical picture led to the diagnosis of opsoclonus-myoclonus syndrome. No neuroblastoma was found by abdominal ultrasound, chest X-ray and meta-iodo-benzylguanidine scintigraphy. Due to positive polymerase chain reaction (PCR) for adenovirus type C subtype 3 in serum (2800 copies/ml) on day 3 after admittance, a parainfectious OMS was suspected. Repeated CSF analysis on day 24 showed persisting inflammatory changes, including 6 leucocytes/µl, positive oligoclonal bands and elevated neopterin-levels. Virological analysis revealed an elevated specific antibody index (ASI) of 5.2 for mumps IgG. Serum antibody testing and PCR ruled out acute infection with mumps, herpes simplex, influenza, parainfluenza, measles, human herpes virus type 6, varicella-zoster and Epstein-Barr virus.

**Fig. 1:**
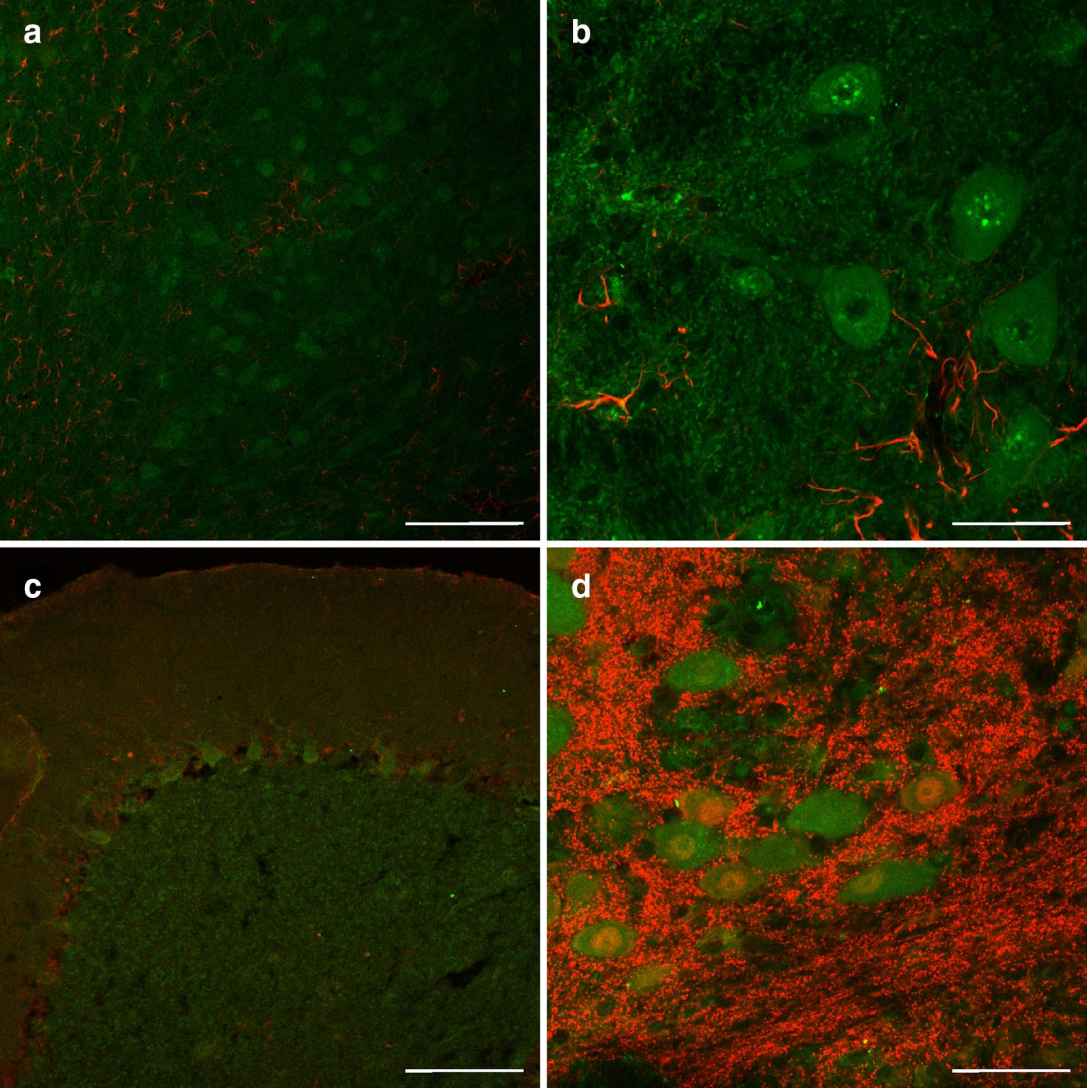
Double immunofluorescence labeling of cerebellar frozen sections from a human autoptic case. Binding sites for autoantibodies from the patient (*green*) and astroglial or neuronal markers (*red*) are concomitantly revealed by confocal laser-scanning microscopy. Autoantigens are stained by patient’s serum (1:20) and carbocyanine (Cy) 2-conjugated anti-human IgG + IgM, while astroglia and neurons are visualized by appropriate Cy3-coupled secondary antibodies. **a** At lower magnification, the cerebellar subcortex displays layers of granular cells immunoreactive for the patient’s serum—clearly distinguishable from immunoreactivity for rabbit-anti-glial fibrillary acidic protein (GFAP; Dakocytomation; 1:1000). **b** At higher magnification, autoantibodies bind cells of deep cerebellar nuclei lacking GFAP immunolabeling. **c** In the cerebellar cortex, Purkinje cells are stained by patient’s serum while rabbit-anti-S100β (Swant; 1:500) predominantly demonstrates protoplasmic astroglia. **d** Immunodecoration of probably neuronal surface antigens with patient’s serum is located apart from labeling achieved with mouse-anti-neuronal nuclei (NeuN; Millipore; 1:100) in the layer of deep cerebellar neurons. *Scale bars* in **a**, **c** = 200 µm, in **b**, **d** = 50 µm

Serum from the patient was applied to indirect immunofluorescence labeling and stained cerebellar neurons (Fig. 1). In parallel, attempts based on onconeural autoantibodies, recognizing the markers Hu, Yo or Ri, and antibodies directed against gangliosides produced no immunolabeling of the same tissue.

Ten weeks after patient’s admittance, immunosuppressive therapy started with methylprednisolone (20 mg/kg) over 5 days every month; after 3 months, the therapy was extended with adrenocorticotropic hormone (ACTH, 60 units three times monthly) and intravenous immuno globulin (IVIG, 1 g/kg every second month) for 1 year. Along with the initiation of this therapy, the neurological status improved.

Due to persisting tremor and irritability, therapy was switched to oral dexamethasone (12 mg each on 3 days monthly) after 1 year and subsequently tapered over 3 years. Overall, the immunosuppressive therapy lasted 50 months.

**Fig. 2:**
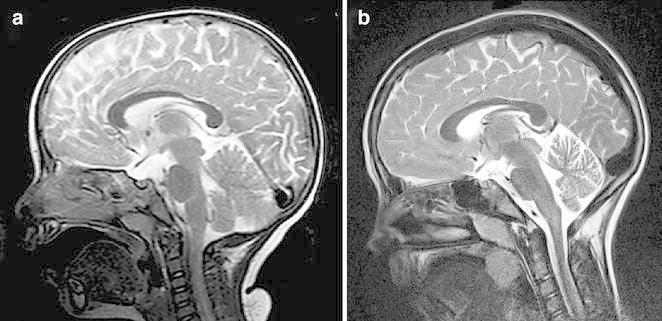
Sagittal T2-weighted cranial magnetic resonance images at onset of opsoclonus-myoclonus syndrome (**a**) and 5 years later (**b**), showing mild cerebellar atrophy with widened sulci and extracerebellar spaces

The patient is now 12 years-old and shows an age-appropriate intelligence, visual-spatial performance and motor performance (Kaufman assessment battery, developmental test of visual perception 2, motoric test for children) and attends a normal primary school. She is infrequently showing slight myoclonic and ataxic movements, worsened during febrile illness. Brain MRI after 5 years was indicative of slight cerebellar atrophy (Fig. 2).

## Discussion and evaluation

Our report illustrates the course of a severe case of OMS. To our knowledge, this is the first patient with an associated adenoviral infection, which was demonstrated by positive PCR during onset of first symptoms. If occult neuroblastoma had been present in this patient it presumably regressed spontaneously. Outcome of OMS is often associated with long-term neurological morbidity and cognitive problems (Klein et al. 2007); however, the long-term but moderately intense immunosuppressive treatment in our patient led to nearly complete recovery with age appropriate cognitive function. Adenoviral infection is a likely cause of OMS in our patient. We could prove the hypothesis of an immune-mediated response to cerebellar neurons by immunohistochemistry, which suggests that OMS is affecting cerebellar neurons. Serum antibodies of our patient stained cerebellar neurons with probably different antigens, as there was positive staining of Purkinje cells, granule cells and neurons of deep cerebellar nuclei. Immunofluorescence labeling predominantly revealed membrane-associated antigens. Despite the lack of a control group, this observation is in line with previous studies, which showed similar immunostaining patterns, but failed to identify defined antigens (Blaes et al. 2005; Connolly et al. 1997).

Another fact confirming a predominant cerebellar inflammation is the brain MRI on 5-year follow-up with mild signs of cerebellar atrophy—a finding previously reported by Hayward and co-workers (Hayward et al. 2001). Apart from a specific immune response to cerebellar neurons, the patient displayed a non-specific humoral immune response in the CNS with up-regulation of vaccination titers, as indicated by an elevated ASI of 5.2 for anti-mumps IgG and oligoclonal bands in CSF. The patient had no history of previous mumps infection and was vaccinated (MMR Triplovax, Sanofi-Pasteur-MSD) 4 months prior to the onset of OMS, which makes a causal relationship unlikely. Another patient with OMS was characterized by elevated anti-rubella-antibodies in CSF, suggesting rubella infection as etiologic factor, but evidence of infection was not given (Denne et al. 2006). It is likely that in OMS a non-specific immune response occurs in the CNS comparable to other immune-mediated inflammatory diseases of the CNS (Jarius et al. 2009). The mean delay of 17 weeks in initiation of treatment is unacceptably long (Tate et al. 2005). Our video-documented study will help child neurologists to recognize this treatable movement disorder and to shorten the time to establish immunosuppressive therapy.

## Conclusions

Our case study illustrates severe symptoms of OMS and the favorable outcome after long-term immunosuppressive treatment. Further studies are needed for the identification of specific antibodies as biomarkers and for the recognition of the disease-specific cerebellar antigens in OMS.

The patient and her parents gave written informed consent and agreement to the publication of this report.

## Additional file

10.1186/s40064-015-1429-1 Video showing patient before onset of disease (First Part), 6 weeks after onset with severe ataxic and myoclonic hyperkinetic movements and opsoclonus (Second Part) and at the age of 8 years demonstrating favorable outcome (Third Part).

## References

[CR1] Blaes F, Fühlhuber V, Korfei M, Tschernatsch M, Behnisch W, Rostasy K, Hero B, Kaps M, Preissner KT (2005). Surface-binding autoantibodies to cerebellar neurons in opsoclonus syndrome. Ann Neurol.

[CR2] Connolly AM, Pestronk A, Mehta S, Pranzatelli MR, Noetzel MJ (1997). Serum autoantibodies in childhood opsoclonus-myoclonus syndrome: an analysis of antigenic targets in neural tissues. J Pediatr.

[CR3] Denne C, Kleines M, Scheithauer S, Heimann G, Häusler M (2006). Methotrexate: a new treatment in opsoclonus-myoclonus syndrome. J Pediatr Neurol..

[CR4] Hayward K, Jeremy RJ, Jenkins S, Barkovich AJ, Gultekin SH, Kramer J, Crittenden M, Matthay KK (2001). Long-term neurobehavioral outcomes in children with neuroblastoma and opsoclonus-myoclonus-ataxia syndrome: relationship to MRI findings and anti-neuronal antibodies. J Pediatr.

[CR5] Jarius S, Eichhorn P, Jacobi C, Wildemann B, Wick M, Voltz R (2009). The intrathecal, polyspecific antiviral immune response: specific for MS or a general marker of CNS autoimmunity?. J Neurol Sci.

[CR6] Klein A, Schmitt B, Boltshauser E (2007). Long-term outcome of ten children with opsoclonus-myoclonus syndrome. Eur J Pediatr.

[CR7] Pang KK, de Sousa C, Lang B, Pike MG (2010). A prospective study of the presentation and management of dancing eye syndrome/opsoclonus-myoclonus syndrome in the United Kingdom. Eur J Paediatr Neurol.

[CR8] Pranzatelli MR, Travelstead AL, Tate ED, Allison TJ, Moticka EJ, Franz DN, Nigro MA, Parke JT, Stumpf DA, Verhulst SJ (2004). B- and T-cell markers in opsoclonus-myoclonus syndrome: immunophenotyping of CSF lymphocytes. Neurology.

[CR9] Rostasy K, Wilken B, Baumann M, Müller-Deile K, Bieber I, Gärtner J, Möller P, Angelini P, Hero B (2006). High dose pulsatile dexamethasone therapy in children with opsoclonus-myoclonus syndrome. Neuropediatrics.

[CR10] Tate ED, Allison TJ, Pranzatelli MR, Verhulst SJ (2005). Neuroepidemiologic trends in 105 US cases of pediatric opsoclonus-myoclonus syndrome. J Pediatr Oncol Nurs.

